# Comparative study of the safety and efficacy of SMOFlipid vs non SMOFlipid as TPN for liver transplantation

**DOI:** 10.1016/j.amsu.2021.01.042

**Published:** 2021-02-18

**Authors:** Mei-Yun Wu, Sheng-Chih Kuo, Su-Fen Chuang, Cheng-Hsi Yeh, Shih-Min Yin, Wei-Feng Li, Hung-Jen Wang, Chao-Long Chen, Chih-Chi Wang, Chih-Che Lin

**Affiliations:** a College of Nursing, Kaohsiung Medical University, Kaohsiung, Taiwan; b Department of Nursing, Chang Gung Memorial Hospital, Kaohsiung, Taiwan; c Department of Surgery, Chang Gung Memorial Hospital, Kaohsiung, Taiwan; d Dietitian, Department of Nutritional, Chang Gung Memorial Hospital, Kaohsiung, Taiwan; e Department of Urology, Chang Gung Memorial Hospital, Kaohsiung, Taiwan

**Keywords:** Liver transplantation, SMOFlipid, Fish oil

## Abstract

**Background:**

Liver transplantation (LT) is one of the widely recognised and leading treatments for end-stage liver disease. Nutrition impacts its success. Total parenteral nutrition (TPN) is usually prescribed for patients recommended prolonged fasting after LT. The supplement of SMOFlipid (soybean oil, MCT oil, olive oil, and fish oil) is easily metabolised to produce energy, and it possesses anti-inflammatory effects; however, SMOFlipid emulsion use raises concerns regarding coagulopathy after LT. This study investigated the postoperative correlation between SMOFlipid and coagulation in LT.

**Materials and methods:**

The medical records of 54 recipients of living donor LT between January 2012 and June 2015 were retrospectively reviewed. Patients with pretransplant platelet count <40,000/μL and >40,000/μL were assigned to the non-SMOFlipid (n = 23) group and the SMOFlipid (n = 31) group, respectively.

**Results:**

The coagulation and nutrition profile of patients improved significantly after TPN support. No significant difference was observed in the coagulation profile between SMOFlipid and non-SMOFlipid groups. Although the SMOFlipid group exhibited a higher platelet count than the non-SMOFlipid group on day 7 (P < 0.001), no significant differences were observed in the platelet count on 14 and 30 days after TPN support between the 2 groups.

**Conclusion:**

TPN using SMOFlipid after LT is a good strategy for improving nutritional status without increasing the risks of bleeding and coagulation in patients intolerant of early enteral nutrition. Moreover, SMOFlipid use may not cause coagulopathy up to 14 days after LT. Overall, SMOFlipid provides nutritional benefits without increasing the risk of bleeding.

## Highlights

• TPN usually prescribed for nutrition support at early stage after liver transplantation is important.
• SMOFlipid emulsion supplement in TPN improves nutritional status during early post-LT period within 14 days.
• TPN support with SMOFlipid emulsion does not impair coagulation parameters nor increase the risk of thrombocytopenia.

## 1 Introduction

Liver failure is characterised by the loss of liver function and is complicated with hepatic encephalopathy and coagulopathy [[1], [2], [3]]. Liver transplantation (LT) is one of the widely recognised and leading treatments for end-stage liver disease [2,4]. Malnutrition is one of the common manifestations of this critical condition and is also an independent predictor of mortality [[3], [4], [5]]. Several studies have shown that malnutrition is a poor prognostic factor for LT, which indicates that nutritional support may reduce LT complications and improve survival [4].

Total parenteral nutrition (TPN) is usually prescribed for patients recommended prolonged fasting after LT. The supplement of SMOFlipid (soybean oil, MCT oil, olive oil, and fish oil) has the advantage of being easily metabolised to produce energy, and it has anti-inflammatory effects [[6], [7], [8]]. The major therapeutic mechanism of fish oil is the attenuation of systematic inflammation, which may decrease the mortality risk in patients with severe injury and sepsis [9,10]. SMOFlipid has been proven to be safe and well tolerated in a wide range of clinical conditions, and it is used as the standard lipid emulsion [11,12]. Moreover, the short-term application of parenteral fish oil with soybean oil not only significantly reduces the parameters of liver damage in the postoperative period but also leads to a more balanced immune response, which may result in the faster resolution of inflammation and recovery [10,13]. However, SMOFlipid emulsion use may be associated with coagulopathy after LT [14]. Early studies have shown that the dietary intake of n-3 fatty acids, which is a component of fat, is associated with antithrombotic effects but increases the risk of bleeding [15]. A detailed analysis is lacking, and these observations have yet to be proven; this concern persists [16,17]. Hence, we should pay attention to the bleeding tendency when using fish oil fat emulsion because it may aggravate the risk of bleeding. Therefore, the use of fish oil-containing fat emulsion and its related risks is a clinically important issue in early LT that should be investigated, because liver function is not restored immediately after transplantation, and there is a tendency of coagulopathy. Thus, classical haemostasis parameters such as activated partial thromboplastin time (aPTT) and platelet count are measured prior to surgery and before the start of TPN. However, many questions remain unanswered regarding nutritional assessment and support for these seriously ill, nutritionally, and metabolically complex patients [13]. This study evaluated the effect of the SMOFlipid supplement in TPN in LT patients.

## 2 Materials and methods

### 2.1 Study design

This study was approved at ClinicalTrials.gov (ID: NCT04572373) and has been reported in line with the STROCSS criteria [18].Adult patients who underwent LT at Kaohsiung Chang Gung Memorial Hospital between January 2012 and June 2015 were screened in this study. The medical records of patients were retrospectively reviewed to check if TPN was provided in the last 14 days in the peri-transplantation period.

TPN and SMOFlipid support were indicated for patients who received NPO for more than 3 days, such as those with repeated laparotomy, staged biliary reconstruction, massive nasogastric (NG) drainage (>500 mL/day), ileus, diarrhoea, poor digestion (NG extraction >50 mL/time), and chylous ascites. Furthermore, SMOFlipid was discontinued when the platelet count decreased to 40,000/μL or less. In all cases, heparinisation was prescribed to maintain the aPTT level between 1.5 and 2 times the normal controlled level at least for 10–14 days, with daily blood examination conducted. Oral administration of dipyridamole (75 mg, QID) for 3 months was indicated for stimulation of antiplatelet activity when the platelet count increased to 40,000/μL or more.

### 2.2 Patient selection

A total of 54 adult (age > 18 years) LT recipients were enrolled, excluding those with renal dysfunction (eGFR < 60 mL/min/1.73 m^2^). On the basis of our experience in patient management at this institute, we allocated patients with a pretransplant platelet count less than 40,000/μL and those with a count more than 40,000/μL to the non-SMOFlipid group (n = 23) and the SMOFlipid group (n = 31), respectively. Patients with well-tolerated oral intake and those in whom the TPN supplement was discontinued within 10 days were excluded from this study.

### 2.3 Operative procedures and postoperative care

The surgical procedures of liver transplantation are described elsewhere [[19], [20], [21], [22]]. After portal vein anastomosis, the graft was re-perfused by consecutively unclamping the hepatic and portal veins. The hepatic artery was subsequently anastomosed, followed by biliary anastomosis. Venous outflow reconstruction was performed by anastomosing the donor and recipient hepatic veins in an end-to-end manner. Hepatic artery reconstruction was performed by a microsurgeon using the microsurgical technique. The types of biliary reconstruction and drainage to be provided to patients were judged by the same microsurgeon according to the diameter, number, and viability of the bile ducts, and the biliary reconstruction and drainage were performed using microsurgery. After transplantation, liver ECHO with Doppler was conducted daily for at least 14 days to detect early vascular complications.

For immunosuppression, methylprednisolone, Tacrolimus, and Mycophenolate mofetil were used. Basiliximab was initiated after portal vein re-perfusion and on postoperative day 4. Tacrolimus treatment was initiated on postoperative day 1, and a trough plasma concentration of 8–10 ng/mL was maintained during the first month and 5–8 ng/mL thereafter. Antithrombotic treatment consisted of heparin and PGE1 use for the first 7–14 days postoperatively.

### 2.4 Data collection

The study patients were divided into the SMOFlipid group (n = 31) and a non-SMOFlipid group (n = 23). Medical records including patient demographics, preoperative history, physical examination, clinical course, laboratory studies, aetiology of liver disease, severity of liver disease including Child–Pugh score and the Model for End-Stage Liver Disease (MELD) score, postoperative complications, and length of ICU and hospital stay were collected and reviewed.

### 2.5 Ethics

This study was performed after approval from the Committee on Ethics in Clinical Research of the Kaohsiung Chang Gung Memorial Hospital, Taiwan (IRB number: 201601322A3). All patients were explained the objectives and possible adverse reactions of the supplement used in the study. Patients provided voluntarily written informed consent prior to their enrolment into the study or before they received supplementation with the fat emulsion.

### 2.6 Statistical analysis

Numerical variables are presented as mean ± SD, unless indicated otherwise. Statistical analyses were performed using SPSS version 22. One-way ANOVA and Scheffe's method were used to analyse changes with time within the treatment groups. The *t*-test was used to compare variables between different time points and to compare the variables between groups at a particular time point. Linear correlations were determined using Pearson's correlation coefficients. P < 0.05 was considered statistically significant.

## 3 Results

A total of 54 patients who underwent LT between 1 January 2012 and 30 June 2015 received TPN. Of them, 49 patients underwent living donor LT, and the other 5 patients underwent deceased donor LT. The total number of deaths within 14 and 30 days of LT were zero and 2, respectively.

### 3.1 Patient demographics and clinical characteristics of SMOFlipid and non-SMOFlipid groups

Patient demographics and clinical characteristics showed no significant difference between the SMOFlipid and non-SMOFlipid groups in terms of aetiology and severity of liver disease (Child–Pugh classification and MELD score). The non-SMOFlipid group had a longer length of hospital stay than the SMOFlipid group; however, no significant difference was observed (Table 1).

**Table 1:** Demographics and clinical characteristics of LT recipients.

Total N = 54	SMOFlipid n = 31	non-SMOFlipid n = 23	P value
Gender (M/F)	17/14	11/12	0.610
Age	53.54 ± 11.77	54.07 ± 10.20	0.863
**Diagnosis**			0.464
HBV	13	5	0.120
HCV	9	8	0.653
Alcoholism	3	3	0.697
others	6	7	0.345
**Severity of disease**			
Child			0.271
A	8	5	0.730
B	12	5	0.184
C	11	13	0.124
MELD score	21.42 ± 8.547	23.11 ± 9.42	0.543
**Complication**			0.581
Surgical complications	13	10	0.910
staged biliary reconstruction	8	7	
bile leak	7	4	
HA stenosis-redo HA	2	2	
Medical complication	11	9	0.784
Prolong intubation	5	1	
delirium	6	5	
Sepsis	5	5	
ACR	3	5	
**Indication of TPN**			0.861
prolonged NPO	14	9	0.658
poor oral intake	13	11	0.910
other	4	3	0.646
ICU stay (day)	27.81 ± 16.67	28.57 ± 18.56	0.875
Hospital stay (day)	89.94 ± 60.89	103.7 ± 81.41	0.480
Admission (times)	2.87 ± 2.67	3.96 ± 2.87	0.158

### 3.2 Improvement of coagulation profile after nutrition support in SMOFlipid and non-SMOFlipid groups

**Fig. 1:**
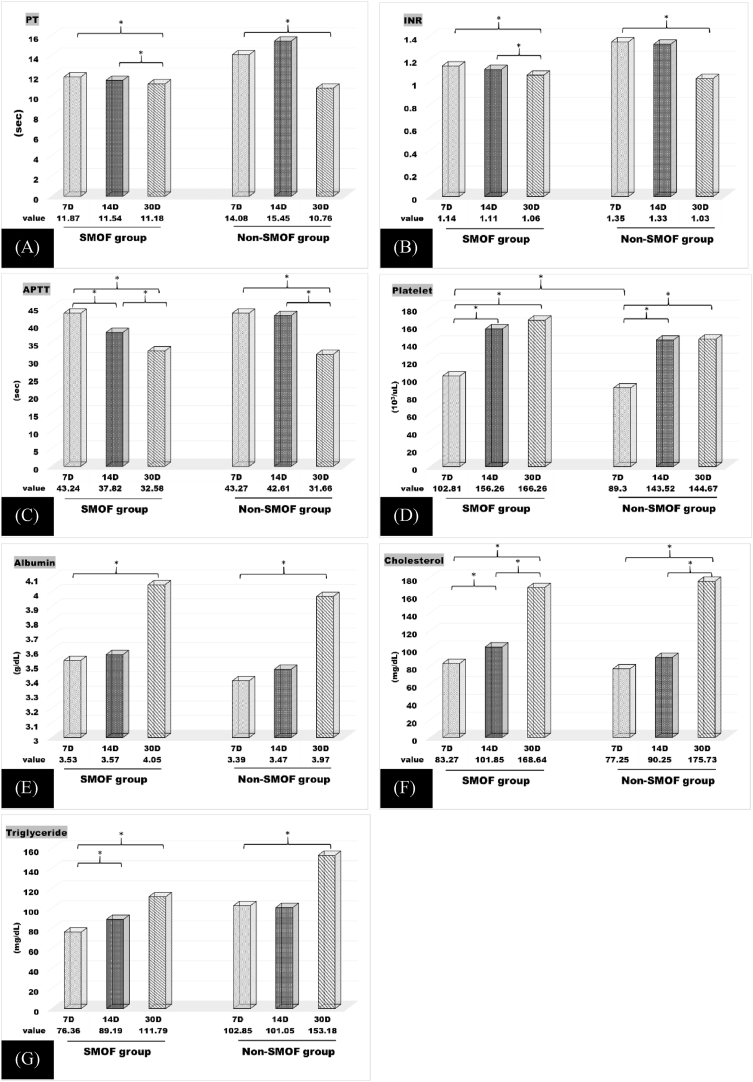
Comparison of coagulation parameters (PT(A), INR(B), APTT(C), Platelet(D)) and nutrition parameters (Albumin(E), Cholesterol(F), Triglyceride(G)) between both groups. The serum level of INR (A) APTT (B) Platelet (C) Albumin (D) Cholesterol and (E)Triglyceride (F) with or without using SMOF; *:significantly different between the two group.

The effects of nutrition support on coagulation statuses are presented in Fig. 1. During nutrition support, all coagulation parameters namely prothrombin time (PT), international normalized ratio (INR), aPTT, and platelet count improved gradually in both the SMOFlipid and non-SMOFlipid groups (Table 2, Table 3). The non-SMOFlipid group exhibited lower platelet count than the SMOFlipid group 7 days after TPN support (Fig. 1); however, no significant differences on 14 and 30 days after TPN support were observed in platelet counts between the groups (Fig. 1).

**Table 2:** Coagulopathy profile and nutrition profile in SMOFlipid group.

viable	7 days vs. 14 days P value	7 days vs. 30 days P value	14 days vs. 30 days P value	Sphericity Assumed
PT (sec)	0.124	0.044*	0.103*	0.033*
INR (sec)	0.060	0.012*	0.042*	0.005*
aPTT (sec)	0.002*	0.0001*	0.002*	0.0001*
Platelet (10^3^/μL)	0.022*	0.0001*	0.187	0.002*
Albumin (g/dL)	0.280	0.046*	0.119	0.045*
Cholesterol (mg/dL)	0.0001*	0.0001*	0.0001*	0.0001*
Triglyceride (mg/dL)	0.006*	0.006*	0.360	0.007*

30 days after nutrition support in SMOFlipid group, all coagulation and nutrition parameters showed significant difference (p < 0.05) when comparing with 7 days after nutrition support.

**Table 3:** Coagulopathy profile and nutrition profile in non-SMOFlipid group.

viable	7 days vs. 14 days P value	7 days vs. 30 days P value	14 days vs. 30 days P value	Sphericity Assumed
PT (sec)	0.347	0.011*	0.064	0.027*
INR (sec)	0.983	0.021*	0.075	0.020*
aPTT (sec)	0.540	0.0001*	0.007*	0.0001*
Platelet (10^3^/μL)	0.023*	0.013*	0.992	0.023*
Albumin (g/dL)	0.101	0.062	0.179	0.063
Cholesterol (mg/dL)	0.018*	0.0001*	0.0001*	0.0001*
Triglyceride (mg/dL)	0.451	0.026*	0.076	0.021*

30 days after nutrition support in non-SMOFlipid group, coagulation and nutrition parameters showed significant difference (p < 0.05) except for albumin level when comparing with 7 days after nutrition support.

SMOFlipid, Soybean oil, MCT oil, Olive oil, Fish oil; PT, Prothrombin Time; INR, International normalized ratio; aPTT, activated Partial Thromboplastin Time.

### 3.3 Improvement of nutrition profile after nutrition support in SMOFlipid and non-SMOFlipid groups

The 3 nutritional parameters, namely albumin, cholesterol, and triglyceride levels, also improved gradually after nutrition support (Fig. 1 and Table 2, Table 3). Cholesterol and triglyceride levels showed no significance between the non-SMOFlipid and SMOFlipid groups. We reported no bleeding events, such as postoperative intra-abdominal bleeding, upper gastrointestinal bleeding, or intra-cranial haemorrhage, in our care group.

## 4 Discussion

Postoperative nutrition support is critical for LT recipients. Our findings indicate that TPN support using SMOFlipid is safe and efficient for improving the nutritional status of patients, and SMOFlipid use does not impair coagulation parameters and does not increase the risk of thrombocytopenia.

The classical hemostatic parameters, such as prothrombin time (PT) aPTT, and platelet counts, were measured before TPN support and on 7, 14, and 30 days after TPN support. In the post-TPN observation period, the coagulation capacity was regained. As hypothesised, no differences were observed in PT, aPTT, and platelet counts. Overall, SMOFlipid administration was shown to be safe without increased coagulation risk and did not influence the platelet count [11,16].

Protein-energy malnourishment and metabolic abnormalities are common in patients with end-stage liver disease who undergo LT [14]. The malnutrition may further increase morbidity, mortality, and costs in the post-transplantation setting [14,23]. Thus, nutritional support during all phases of LT is crucial; although enteral nutrition is preferred, parenteral nutrition may be provided to supply the required calorie intake (14). Therefore, administration of SMOFlipid in the postoperative period can be considered valuable for patients requiring parenteral nutrition after major abdominal surgery [24]. In our series, SMOF lipid as nutrition support after LT showed benefits in the short term. However, it should be investigated whether this intervention has benefits after long-term use.

In addition, the nutritional status in LT recipients can worsen rapidly in the immediate post-transplantation period due to preoperative malnutrition, immunosuppressive therapy, fasting, surgical stress, and postinterventional complications [23,25]. However, nutrition support with lipid supplements also raises the concern of possible adverse effects, such as prolonged bleeding time, thrombocytopenia, coagulation disorders, hyperlipidaemia, and deteriorating liver function [16,17]. These anti hemostatic consequences might lead to bleeding complications, prolonged hospital stay, and surgical mortality after LT. In our series, the application of SMOFlipid improved the nutritional status in LT recipients, with no evident increase in the risks of bleeding, surgical mortality, and thrombocytopenia. Thus, these findings indicate that an aggressive early postoperative nutritional support (via the enteral route, if possible) with lipid-containing agents should be provided to patients with the highest MELD score, without any concern for the bleeding tendency [23].

ω‐3 (n‐3) fatty acids are essential polyunsaturated fatty acids derived from fish oil. The benefits of nutritional supplementation with ω‐3 fatty acids in decreasing postoperative infection complications and length of hospital stay have been proven in patients undergoing elective gastrointestinal surgery, particularly in those with preoperative malnourishment [3,26]. Therefore, post-transplant partial parenteral nutrition support can greatly improve the protein metabolism and nutritional states. A study also showed that ω-3 fatty acid supplementation for parenteral nutrition significantly decreases injury of the transplanted liver, incidence of infectious morbidities, and the post-transplant hospital stay [14,27]. Our study also demonstrated a similar effect of nutrition support with SMOFlipd without the risk of SMOFlipid-related adverse events, such as postoperative bleeding and thrombocytopenia. Therefore, SMOFlipid might provide a safe and efficient nutrition support in critical patient care after LT.

In addition to the effects of lipid emulsions on coagulation and nutritional status, studies have discussed their effects on the inflammatory response. SMOFlipids are enriched with w-3 fatty acids and thus inhibit the production of proinflammatory cytokines [[6], [7], [8]], and they are considered beneficial for patients at risk of inflammation [28,29]. Furthermore, fish oil supplements for surgical [30] and critically ill patients [31] are recommended by the European Society of Clinical Nutrition and Metabolisms. In this study, we only focused on the effects of SMOFlipid supplements on coagulation and nutritional status after liver transplantation in patients. The anti-inflammatory effects were not evaluated in surgical and critically ill patients and warrant investigation.

## 5 Conclusion

TPN after LT is an effective strategy for improving nutrition and coagulation in patients exhibiting early enteral feeding intolerance. Additionally, the use of SMOFlipid may not cause coagulopathy up to 14 days after LT. Thus, SMOFlipid provides nutritional benefits without increasing the risk of bleeding.

As shown previously, the application of intravenous SMOFlipid as a component of the mixed type emulsion is not associated with any negative effects including the risk of coagulopathy. Furthermore, the significance of carefully assessing the nutritional status during the work-up for patients who are candidates for LT is widely recognised. Additionally, this approach should be considered when patients cannot tolerate oral intake. On the basis of our findings, for patients with platelet counts higher than 40,000/μL, although the nutritional status after SMOFlipid supplements was similar to that in the non-SMOF lipid group, the administration of SMOFlipid TPN was safe, with no evident coagulopathy or worsening thrombocytopenia.

### 5.1 Limitation

In this study, we enrolled only 54 patients of the 147 patients who underwent TPN support after LT between 1 January 2012 and 30 June 2015. Furthermore, the retrospective design of the study may have affected the quality of the obtained data. In addition, we excluded patients with extreme thrombocytopenia (platelet count <40,000/μL), and the anti-inflammatory effects of SMOFlipid were not assessed in this study. Furthermore, coagulopathy and thrombocytopenia might be associated not only with nutrition supplementation but also with other clinical conditions, including medication usage, graft quality, sepsis, and other complications, in LT recipients. Moreover, the components of SMOFlipid are complicated; determination of the individual effects of each component, such as ω‐3 or ω‐6 fatty acids, on LT recipients requires further analysis. Further larger, prospective, randomised studies are required to determine whether SMOFlipid provides benefit in terms of both nutrition and postoperative recovery in LT recipients.

## Grant information

This work was supported by Grant CMRPG8F0971, CMRPG8F0972, CMRPG8F0981 and CMRPG8F0982 from the 10.13039/100012553Chang Gung Memorial Hospital, R.O.C.

## Author contribution

Mei-Yun Wu; concept of study, data collection, data analysis, writing paper, Sheng-Chih Kuo; concept of study, writing paper, Su-Fen Chuang; data collection, Cheng-Hsi Yeh; data collection, Shih-Min Yin; data collection, Wei-Feng Li; data collection, Hung-Jen Wang; data collection, Chao-Long Chen; concept of study, Chih-Chi Wang; concept of study, Chih-Che Lin; concept of study, critical manuscript.

## Registration of research studies

Research Registry Unique Identifying Number: ClinicalTrials.gov, Registration ID: NCT04572373.

Hyperlink to my specific registration: https://register.clinicaltrials.gov/prs/app/action/LogoutUser?uid=U0002IDV&ts=34&cx=ubqdhy.

## Guarantor

Wipusit Taesombat.

## Provenance and peer review

Not commissioned, externally peer reviewed.

## Declaration of competing interest

All of authors in this study declared that there were no conflicts of interest.
